# Perspectives on CRISPR Genome Editing to Prevent Prion Diseases in High-Risk Individuals

**DOI:** 10.3390/biomedicines12081725

**Published:** 2024-08-01

**Authors:** Milan M. Medd, Qi Cao

**Affiliations:** Feinberg School of Medicine, Northwestern University, Chicago, IL 60611, USA

**Keywords:** neurodegenerative disease, prion disease, CRISPR, genome editing, prevention

## Abstract

Prion diseases are neurodegenerative disorders caused by misfolded prion proteins. Although rare, the said diseases are always fatal; they commonly cause death within months of developing clinical symptoms, and their diagnosis is exceptionally difficult pre-mortem. There are no known cures or treatments other than symptomatic care. Given the aggressiveness of prion diseases on onset, therapies after disease onset could be challenging. Prevention to reduce the incidence or to delay the disease onset has been suggested to be a more feasible approach. In this perspective article, we summarize our current understandings of the origin, risk factors, and clinical manifestations of prion diseases. We propose a PCR testing of the blood to identify *PRNP* gene polymorphisms at codons 129 and 127 in individuals with familial *PRNP* mutations to assess the risk. We further present the CRISPR/Cas9 gene editing strategy as a perspective preventative approach for these high-risk individuals to induce a polymorphic change at codon 127 of the *PRNP* gene, granting immunity to prion diseases in selected high-risk individuals, in particular, in individuals with familial *PRNP* mutations.

## 1. Introduction

Misfolded prions are unique infectious agents composed entirely of protein [1]. Unlike other pathogens, prions lack nucleic acids and consist solely of misfolded protein forms. The term “prion” is derived from “proteinaceous infectious particles” [2]. Normal mammalian tissues ubiquitously express the non-pathogenic form of the prion protein (PrP^C^), with particularly high expression in the central nervous system (CNS) [3]. PrP^C^ is expressed on the cell surface and is implicated in key cellular processes including cell signaling and adhesion, neurogenesis, and neuronal homeostasis [4,5,6,7,8]. Prion diseases, also known as transmissible spongiform encephalopathies (TSEs), are subacute and fatal disorders that result in the neurodegeneration of the central nervous system (CNS) [5]. Common TSEs include Creutzfeldt–Jakob disease (CJD) and kuru in humans, scrapie in sheep, and bovine spongiform encephalopathy (BSE) in cows [9]. Here, we provide a concise review of our current understanding of the molecular genesis of TSEs and propose a perspective preventative approach to eliminate or reduce the prevalence of prion diseases in high-risk individuals.

**Figure 1:**
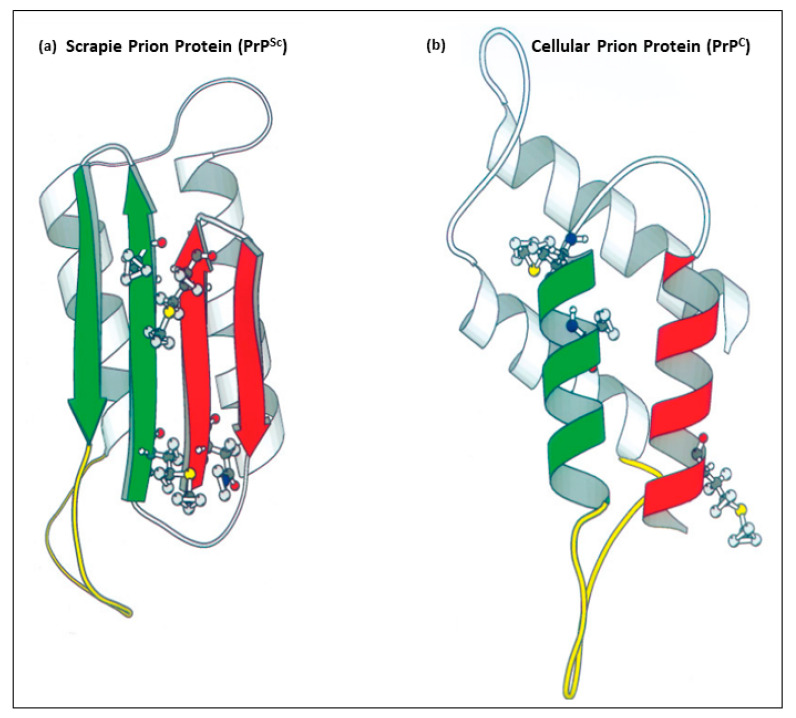
Images of the tertiary structure of human prion proteins. (**a**) is the pathogenic protease-resistant misfolded scrapie prion isoform (PrP^Sc^) with a high abundance of β-pleated sheets. (**b**) is the normal cellular prion protein (PrP^C^) with an α-helical structure. (**a**,**b**) are adapted from Huang et al. [26].

TSEs are caused by a misfolded isoform of PrP^C^ known as the scrapie prion protein (PrP^Sc^), although the exact mechanisms behind prion misfolding are not entirely understood and are an active subject of ongoing research [10]. PrP^Sc^ is highly prone to aggregation and can induce the misfolding of additional PrP^C^ molecules, leading to the formation of neurotoxic amyloid plaques and fibrils in the brain [11,12]. This pathogenic feature of PrP^Sc^ gave these disorders the name “Prion Diseases”. Structural comparisons demonstrated that the normal prion protein PrP^C^ has a pronounced α-helical structure, while the pathogenic prion protein PrP^Sc^ has an exceedingly high abundance of, nearly all, *β*-sheets [13,14,15] (Figure 1). The conformational changes in PrP^Sc^ make it resistant to proteolytic degradation, further promoting plaque and fibril formation [16]. In addition to their protease-resistant properties, TSEs are characterized by their inability to elicit a detectable immune response [17,18]. Unlike most diseases, the infectious agent PrP^Sc^ is a protein peptide that has an identical amino acid sequence to the normal cellular protein isoform PrP^C^ [19]. This feature prevents the immune system from eliciting active immune responses against the misfolded PrP^Sc^ particles [4,5,20,21,22]. Therapeutic approaches, such as targeting PrP^Sc^-encoding nucleic acids or monoclonal antibodies targeting PrP^Sc^ are challenged by its identical peptide sequence to PrP^C^ and the critical role of PrP^C^ in normal cellular physiology [23]. Moreover, the lack of effective treatments and early diagnostic markers often results in a non-definitive diagnosis at an advanced stage of the disease, further complicating efforts to manage or cure TSEs [24]. With the lessons from the kuru epidemic and our understanding of the human *PRNP* gene polymorphisms at codon positions 127 and 129 [25], we propose a perspective preventive approach utilizing CRISPR/Cas9 technology to induce *PRNP* gene polymorphism at codon positions 127 and 129 to provide resistance to prion diseases in high-risk individuals. Given the infancy stage of the CRISPR technology, we also discuss the current challenges of utilizing this technology in humans.

## 2. Current Understandings of Human TSEs

Prion diseases can be sporadic, genetic, or acquired [5]. Sporadic TSEs make up approximately 85% of prion diseases in humans, identified by the spontaneous conversion of PrP^C^ into PrP^Sc^ with unknown cellular drivers [5,25]. Genetic TSEs are suspected to occur when the autosomal dominant mutation in the *PRNP* gene is inherited by children of whom at least one parent is a carrier [27,28]. Genetic TSEs contribute to 9–15% of human cases [29]. Acquired prion diseases have foodborne transmission and can be spread either zoonotically or between humans [30]. Other sources of acquired TSEs are the iatrogenic inoculation from prion-contaminated medical devices, such as surgical instruments and electrodes, and blood transfusions [31].

Human TSEs can present as kuru, Creutzfeldt–Jakob disease (CJD), Gerstmann–Sträussler–Scheinker syndrome (GSS), and fatal insomnia (FI), with the majority being CJD [32,33]. The most notable forms of CJD are sporadic CJD (sCJD) and variant CJD (vCJD), though they can also be transmitted genetically as familial CJD (fCJD) or iatrogenically (iCJD) [32,33,34]. sCJD contributes to approximately 85% of human prion diseases [5]. The precise drivers or risk factors for sCJD are not clear. However, methionine homozygosity at codon 129 (Met^129^/Met^129^) in the *PRNP* gene is one of the recognized high-risk factors for sCJD [35]. Data from three series studies in nine countries revealed that a mutation of Met^129^ contributes to at least 70% of the sCJD cases [35,36,37]. Age is also a risk factor for the Met^129^ mutation, with advanced ages being more susceptible [38]. vCJD is a zoonotic form that is contracted through the consumption of cattle affected by bovine spongiform encephalopathy (BSE), a prion disease that affects cows commonly referred to as “Mad Cow Disease” [39,40].

sCJD primarily exhibits clinical symptoms later in life, appearing in individuals between 55 and 75 years old [41,42]. Although the subclinical phase can be years to decades, the disease rapidly progresses on onset and is always fatal, with approximately 90% of patients dying within a year after the non-specific onset of symptoms [41,42]. While symptoms are highly variable, patients may initially exhibit vertigo, fatigue, insomnia, and headache [43]. These symptoms can be accompanied by memory loss, behavioral changes (depression, irritability, apathy, mood swings), and sensory changes (incoordination, visual impairment) [43]. The late stages of the disease have the potential to cause cerebellar ataxia, myoclonus, and more pronounced dementia and disorientation [43]. Extrapyramidal symptoms include bradykinesia, dystonia, rigidity, and possibly blindness [43]. As the disease continually develops, patients can gradually lose speech and mobility leading up to their death [43]. Secondary infections, such as pneumonia, are potential causes of death in TSEs [43]. vCJD, unlike sCJD, usually occurs in younger individuals, with the median age of death being 28 years old [43]. vCJD also has a longer subclinical phase than sCJD of 13–14 months [44]. The initial symptoms are similar to those of sCJD, but the most prominent early signs are painful sensory symptoms including dysesthesia and paresthesia [44,45,46].

PrP^Sc^, due to its tertiary and/or quaternary structure, is characterized by its distinctive properties, especially the ability to maintain its integrity under conditions that would denature most other proteins [16]. This stability allows prions to accumulate in cells. The scrapie isoform is protease resistant and is highly heat resistant. Cooking meat infected with PrP^Sc^ may reduce, but will not completely eliminate, its pathogenicity [16,47]. It was shown experimentally that heating PrP^Sc^ at 600 °C and 1000 °C can achieve lower infectivity and destroy prion infectivity, respectively [48]. In laboratory practices, heating to 115 °C or autoclaving was shown to be effective in reducing the infectivity of prions causing BSEs [47]. Certain proteases can degrade PrP^Sc^, but the optimum conditions for degradation are a high pH of 10–12 and a temperature of 50–60 °C [48,49]. This provides a solution for destroying prions in the environment but is not feasible in vivo or during food processing. Based on a series of experiments conducted by the CDC, autoclaving PrP^Sc^-contaminated instruments in sodium hydroxide is sufficient to eliminate the risk to the operator [50]. The CDC has developed a guideline that is incorporated in “Chemical and Autoclave Sterilization Methods Outlined in Annex III of the WHO Infection Control Guidelines for TSEs”.

**Figure 2:**
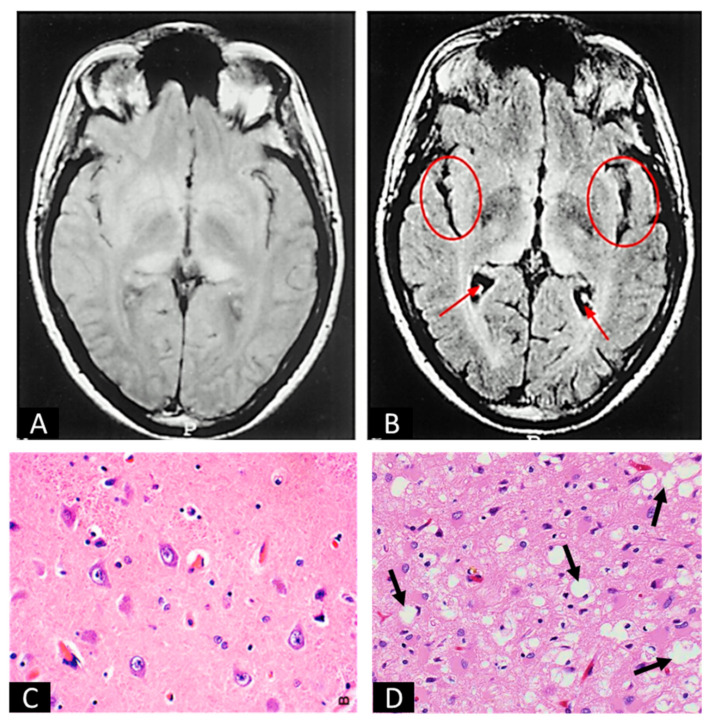
Images of uninfected and prion-infected brain tissues. (**A**,**B**) are MRI scans of uninfected (**A**) and prion-infected (**B**) brains. Arrows and circles in (**B**) indicate the gross images of spongiform degeneration in two regions of the brain. (**C**,**D**) are H&E stains of uninfected (**C**) and prion-infected (**D**) brain sections. Arrows in (**D**) indicate spongiform degeneration at the cellular level. (**A**,**B**) are adapted from Zeidler et al. [67]. (**C**) is from Practical Surgical Neuropathology: A Diagnostic Approach (Second Edition by Daniel J. Brat), Chapter 2, Normal Brain Histopathology [68]. (**D**) is adapted from Britannica, T. Editors of Encyclopaedia [69].

The mechanisms by which PrP^Sc^ infects and degrades the CNS are not well understood. Notwithstanding, enough information is present to draw plausible inferences about acquired PrP^Sc^. It is believed that orally acquired PrP^Sc^ exploits the gut-associated lymphoid tissue (GALT) in the small intestine as a means to spread to the CNS [51]. The GALT typically functions with the mesenteric lymph nodes (MLNs) to prevent infections by triggering the production of immune cells [51]. Intestinal M cells, a type of intestinal epithelial cell (IEC) located in the same region as GALT lymphoid follicles, usually function to translocate antigens to the GALT to elicit an immune response [52]. In the case of PrP^Sc^ infections, the M cells uptake the PrP^Sc^ protein into the GALT and spread into follicular dendritic cells (FDCs), specialized antigen-presenting cells in the lymphoid follicles [53]. PrP^C^ is a cell-surface glycoprotein attached to the plasma membrane by a glycosylphosphatidylinositol (GPI) anchor found in high levels in lymphoid cells in addition to the CNS in which it is prominent. Thus, the protein is also largely expressed in the plasma membrane of FDCs [54,55,56,57,58]. As FDCs capture and retain PrP^Sc^, the high concentrations of PrP^C^ enable the replication and accumulation of PrP^Sc^ by inducing the misfolding of PrP^C^ [53,56]. When the concentration is sufficient for neuroinvasion, PrP^Sc^ spreads, possibly via the peripheral nervous system, to infect the CNS [53]. The hematogenous spread of prions to the CNS may also be possible [53]. Whether non-acquired CJD utilizes the same neuroinvasion pathways or directly occurs in the CNS is not clear. Once PrP^Sc^ occurs in the CNS, it is suspected to spread between cells by means including direct cell contact by tunneling nanotubes (TNTs), exosome-mediated transfer, cell-to-cell contact, or from membrane budding and transport in vesicles [5]. After dissemination, PrP^Sc^ induces abnormal conformational changes in the already-formed PrP^C^ of surrounding cells, resulting in an exponential rate of PrP^Sc^ replication and aggregation [5]. PrP^Sc^ accumulation causes spongiform degeneration, neuroinflammation, neuronal apoptosis, and synaptic changes [59] (Figure 2). How prion protein aggregates cause neurodegeneration is not well understood [59].

Prion diseases are exceedingly difficult to diagnose, as they have incubation periods of years to decades and exhibit clinical symptoms that are indistinguishably similar to other neurological disorders [24]. Furthermore, all definite diagnosis methods require a sample of brain tissue that can only be extracted postmortem [24]. Testing methods include the processing of tissue using immunohistochemistry or Western blotting for PrP^Sc^ detection [24]. Antemortem testing using EEGs, MRIs, and examining cerebrospinal fluid for elevated 14-3-3 protein levels can be conducted but is limited to a non-definitive diagnosis [60,61,62]. Real-time quaking-induced conversion (RT-QuIC) assays have made a considerable impact on clinical diagnosis and have become the standard tool for sCJD diagnosis [63,64,65,66]. By exploiting the ability of PrP^Sc^ found in cerebrospinal fluid (CSF) to induce the conversion of PrP^C^ to PrP^Sc^ and the consequent aggregation, the RT-QuIC assay can detect the formation of the aggregated PrP^C^ in real time using fluorescent dyes [63,64,65,66]. The method can reach a specificity of 99–100% and sensitivities of up to 97%, depending on the alleles being detected of MM, MV, or VV and specific study sites [63].

Certain individuals have a higher risk of developing sCJD, and the relative risk factors can be determined by examining the polymorphisms at codon 129 in the *PRNP* gene with PCR [70]. The common alleles at this codon are methionine 129 (Met^129^) and valine 129 (Val^129^) [70]. Individuals with homozygotes of the allele Met^129^ (Met^129^/Met^129^) have a higher risk of developing sCJD than those with the heterozygous allele (Met^129^/Val^129^) [70]. Whether individuals with homozygous valine 129 (Val^129^/Val^129^) have a significantly higher risk than those with the heterozygous allele (Met^129^/Val^129^) is not clear, as studies from different historical periods and different geographic locations reported inconsistent outcomes [38,71]. The exact mechanism behind prion protein misfolding in sCJD is still not well understood. Family history also plays a significant role in determining the risk of a patient developing fCJD. Being an autosomal dominant mutation, it is very probable that the children of an fCJD carrier may exhibit clinical symptoms later in life [28].

## 3. Lessons from the Kuru Epidemic

Kuru, a TSE acquired from consuming prion-infected human brain and nervous tissue, was a prominent disease of the Eastern Highlands Province (EHP) of Papua New Guinea in the 1950s before the country conformed to Western influence [72,73,74,75,76,77,78]. A common practice by the Fore tribe, inhabitants of the area, was the consumption of deceased relatives at mortuary feasts to free the spirit of the dead (endocannibalism). It was believed by the Fore tribe that the deceased being eaten by their loved ones was preferable to being consumed by insects. This practice enabled kuru to widely spread among the Fore people, peaking with mortality rates of up to 3.5% of the population during the 1940s and 1950s [79]. The disease disproportionately affected more women and children than men, as it was customary for the women and children to consume the brain while the men preferred muscle tissue. It is noteworthy that 60% of kuru cases were in adult females while only 2% were found in adult males, with the remainder being in children [73,79]. By 1960, the practice of endocannibalism mostly ceased; subsequently, kuru cases gradually disappeared [73].

During the kuru epidemic in the EHP, G127V, a new *PRNP* polymorphism that substitutes glycine (Gly^127^) with Val^127^ arose [80]. The combination of the G127V mutation with homozygous methionine 129 (Gly^127^Met^129^/Val^127^Met^129^) provides complete resistance to kuru and sCJD but not vCJD [80]. With this knowledge, Asante et al. generated transgenic mice that were homozygotes or heterozygotes of human PrP Val^127^Met^129^ (HuPrP Val^127^Met^129^/Val^127^Met^129^ or Gly^127^Met^129^/Val^127^Met^129^) and tested their resistance to human TSEs, including kuru, sCJD, and iCJD. The Val^127^Met^129^/Val^127^Met^129^ mice were completely resistant to all four of the tested human TSE types (types 1–4) by molecular classification [80,81], with no clinical symptoms or subclinical infection [80]. That study also found that mice with the G127V heterozygosity Gly^127^Met^129^/Val^127^Met^129^ were only resistant to human TSE types 1–3 but not to type 4. Whether Val^127^Met^129^/Val^127^Met^129^ or Gly^127^Met^129^/Val^127^Met^129^ provide resistance to other types of human TSEs is not known from this study. X-ray crystallography and NMR spectroscopy demonstrated that the G127V polymorphism induces structural changes that constrain the conformation of PrP^C^ in regions associated with prion disease, preventing β-sheet formation [15,82]. The mutation increases the intermolecular hydrogen bonding between PrP^C^ dimers, reducing the instability and the likelihood of protein misfolding [15,82].

## 4. Current Development in Treating Prion Diseases

The current treatment for prion diseases is limited to supportive care, as no treatments with disease-modifying effects are currently available. With the understanding that PrP^C^ protein misfolding is the cause of prion diseases, various strategies are in development to prevent PrP^C^ misfolding or to target misfolded PrP^Sc^ aggregates. These strategies are often coupled with treatments that directly reduce the production of PrP^C^ [83,84]. However, the development of effective treatments for prion diseases is particularly challenging compared to the more common, slowly progressive neurodegenerative disorders due to the rapid progression and lack of definitive screening biological markers [85]. Furthermore, designing formal clinical trials with an adequate patient population for prion diseases has proven to be extremely difficult, largely because of the rarity of prion diseases. Much of the clinical evidence regarding treatment effects is based on anecdotal observations rather than on rigorous scientific data. Consequently, only six treatments for prion diseases were advanced to the stage of clinical evaluations: flupirtine, quinacrine, doxycycline, pentosan polysulfate, anti-PrP^Sc^ monoclonal antibody PRN100, and a *PRNP* antisense oligonucleotide (ASO) [25,85].

Flupirtine is a triaminopyridine that exhibits anti-apoptotic activity against PrP^Sc^ and amyloid beta (Aβ) peptides [86]. It was the first anti-prion drug to enter clinical trials in which patients with sCJD were treated. While improved cognitive performance was observed, there was no significant improvement in patient survival times [87]. Alternately, quinacrine is an antimalarial drug that was thought to inhibit the conversion of PrP^C^ to PrP^Sc^ [88]. Quinacrine was tested in multiple clinical trials, but the outcomes were inconclusive [89,90,91]. At best, a pilot clinical trial demonstrated that quinacrine induced a marginal improvement in alleviating symptoms [92]. However, expanded clinical trials with larger patient populations did not demonstrate the same benefit of quinacrine [93,94].

With the positive outcome of antibody-based therapy in treating certain neurological disorders such as Alzheimer’s disease [95,96], the use of a monoclonal antibody to target PrP^Sc^ had been pursued. However, clinical trials with the PrP^Sc^-targeting monoclonal antibody PRN100 failed to slow down prion disease progression, despite a reduction in PrP^Sc^ deposits that was found in one patient in a postmortem autopsy [97]. Achieving a plausible clinical benefit with PrP^Sc^-targeting monoclonal antibodies remains challenging.

Tetracyclines, such as doxycycline, have been shown to mitigate PrP^Sc^ aggregation and neurotoxicity and prolong the survival of animals with prion diseases [98,99,100]. Doxycycline has been tested in multiple clinical trials, but conflicting results were observed. In one study, doxycycline was used to treat CJD patients and showed an improvement in patient survival by approximately 80% [101]. Conversely, a different clinical trial showed no significant changes to patient survival and disease progression [102]. The anti-*PRNP* ASO, ION717, was recently developed. ION717 was shown to induce a significantly prolonged survival of mice with scrapie [103]. ION717 recently entered a phase I/II clinical trial (NCT06153966).

## 5. Perspective Prevention Strategies

Although passive and active immunization strategies and RNAi gene therapies to target PrP^C^ have been proposed and are currently in development [25,104,105,106,107,108,109,110,111], given that PrP^C^ is important for normal cellular function, the potential off-target unwanted toxicity could be a concern [112,113,114,115,116]. In another critical aspect, due to the rapid progression of prion diseases once the symptoms begin, achieving clinical benefit within this therapeutic window can be extremely challenging. Thus, preventing or delaying prion disease onset has been suggested as one of the most promising goals in carriers of pathogenic mutations in the *PRNP* gene [25,111].

**Figure 3:**
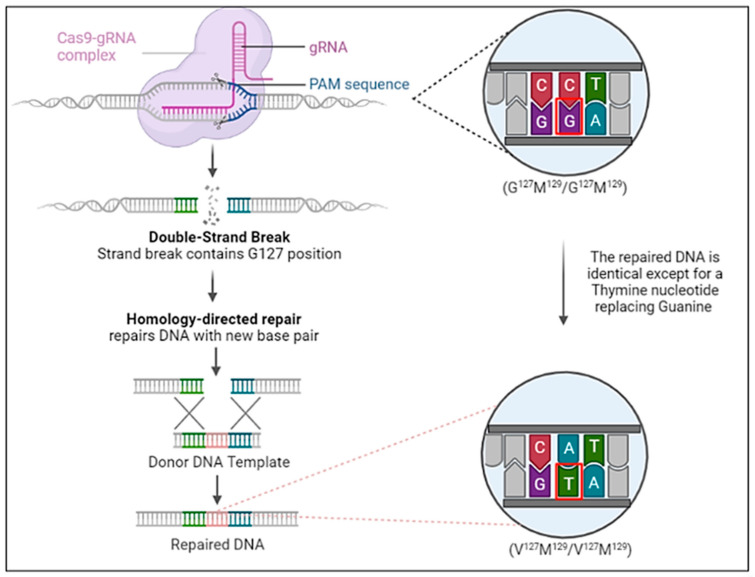
The proposed strategy of CRISPR/Cas9 to introduce the Gly^127^ polymorphism in *PRNP* gene to prevent prion diseases during early life of high-risk individuals, in particular of individuals with familial *PRNP* mutations. Only a single or double base pair substitution (outlined in red) is necessary to induce the Gly^127^ polymorphism into the *PRNP* gene.

With the lessons from the kuru epidemic and the outcome from the transgenic animal study by Asante et al. [80], we propose that CRISPR/Cas9 may be utilized to prevent disease onset in high-risk individuals suspected of being carriers of prion diseases (Figure 3). This proposed approach could leverage the future success of safer viral-vector-based or non-viral delivery systems into the somatic cells of individuals confirmed as being *PRNP* high-risk mutation carriers [117,118,119,120,121,122]. Candidates would be those who have a family history of fCJD and potentially a family history of sCJD with known pathogenic mutations in the *PRNP* gene. Specifically, those with homozygosity for Met^129^ (Gly^127^Met^129^/Gly^127^Met^129^) would be reasonable candidates through PCR screening. The strategy would be introducing one- or two-nucleotide substitution(s) in the chromosome of the 20th pair to induce the desired polymorphic change to Gly^127^Met^129^/Val^127^Met^129^ or Val^127^Met^129^/Val^127^Met^129^ in the *PRNP* gene. gRNA designed to target a region of the *PRNP* gene that includes the 127th codon position functions with the Cas9 enzyme in the Cas9-gRNA complex to induce a double-strand break (DSB) in the targeted area. Noteworthily, the success of CRISPR/Cas9 in preventing TSEs relies on the homology-directed repair (HDR) pathway of the DSB using a donor DNA template with a nearly identical nucleotide sequence. PCR can be used as verification that the mutation has been correctly incorporated after the DSB is repaired. It should be emphasized that the proposed strategy represents a perspective concept or direction in developing feasible strategies for preventing TSEs in high-risk individuals. Acknowledging the potential and the infancy stage of using CRISPR technology in treating human diseases, the feasibility and safety of this proposed strategy need to be rigorously tested in relevant pre-clinical models. Encouragingly, the utilization of CRISP-Cas9 technology to treat human diseases is under active investigation in various pre-clinical animal models of human diseases [123,124,125,126,127,128,129]. Recent clinical trials have demonstrated excellent safety profiles using in vivo CRISPR/Cas9 editing of the *KLKB1* gene to treat patients with hereditary angioedema and editing the *HSV-1* gene to treat patients with herpetic stromal keratitis [130,131]. The FDA approval and later successes of using CRISPR/Cas9 ex vivo genome-edited hematological stem cells to treat sickle-cell disease also shed light on the viability of the proposed approaches [132].

## 6. Current Limitations and Challenges of Using CRISPR Gene Editing for Therapy

Given the recent history of CRISPR/Cas9 technology, its application in treating human diseases is still in its infancy, pending the resolution of the following challenges. The first challenge is potential non-specific integration. At present, CRISPR/Cas9 has the potential to induce nucleotide base mismatches between the gRNA and non-target sequences that may lead to off-target effects, primarily being unknown mutations [133,134,135]. This is caused by a duplex conformation that forms upon mismatched base interactions under strong force [136,137]. Many active investigations are being conducted to overcome these challenges, potentially through approaches to control Cas9 activation. For example, the multiple high-fidelity mutants of Cas9, such as HypaCas9, Cas9-HF1, and SuperFi-Cas9, have exhibited improved accuracy, although at the cost of reduced efficiency [138,139,140,141]. While these mutants are not without limitations, they suggest that utilizing Cas9 variants represents a promising approach for mitigating the off-target effects of CRISPR/Cas9.

The second challenge is the dependency of Cas9 activity on a suitable PAM site to perform base changes [142,143]. This limits the applicability of CRISPR/Cas9 as a therapeutic approach to certain diseases. However, Walton et al. mutated the amino acid sites of Cas9 to produce an SpCas9 variant (SpRY) that is less dependent on PAM restrictions [144]. When inducing the same mutations in the Cas9-HF1 variant (SpRY-HF1), almost all off-target effects were eliminated [145]. Further, modifying Nme2Cas9, a Cas9 variant derived from *Neisseria meningitidis*, has shown promises [146,147]. Nme2Cas9 has strong gene-editing activity in mammalian cells and exhibits enhanced capabilities to target a greater abundance of potential target sites [146,147]. The smaller size of Nme2Cas9 also provides a greater potential for targeted delivery [146,147]. These approaches, although still under active investigations, suggest that developing PAM-independent Cas9 may increase the potential for precise genome editing across a wider variety of genomic sequences.

The third challenge is the potential to induce chromosomal disorganization when repairing DSBs. Activation of the NHEJ repair pathway is the commonly triggered response to DSBs. While the probability of chromosomal disorganization during DSB repair is extremely low, base pair deletions or chromosomal structural translocations can potentially lead to malignancy [148,149,150,151,152,153]. It was shown that the recurrent cleavage of target genes by CRISPR/Cas9 is a significant contributor to the potential occurrence of chromosomal translocations and deletions [148]. It is noteworthy that Yin et al. combined the structural domain of the exonuclease TREX2 with Cas9 to create Cas9TX, which was shown to be highly effective in reducing chromosomal translocations [148]. Collectively, these studies suggest that modifications to Cas9 have the potential to mitigate adverse effects while ensuring genome editing efficiency.

Lastly, the proper delivery systems for clinical use remain an actively investigated area in gene therapy. Both viral and non-viral vectors have been used as the delivery system in clinical studies [154,155]. Improving the specificity of target cell delivery, the efficiency of delivery, and reducing immunogenicity are among the current enduring efforts to improve the delivery system for CRISPR/Cas9 [156,157,158,159,160]. In addition, confounders such as sex, age, and genetic ethnicity should also be considered as crucially important variables that may impact the efficacy of CRISPR/Cas9-based interventions [161]. Notwithstanding, the recent successes proving the safety of using CRISPR/Cas9 in clinical trials, along with the current active investigations to improve the technology, strongly suggest that the future of using this technology to prevent prion diseases in high-risk individuals holds great promise.
